# The Leptin Axis and Its Association With the Adaptive Immune System in Breast Cancer

**DOI:** 10.3389/fimmu.2021.784823

**Published:** 2021-11-15

**Authors:** Laura García-Estevez, Silvia González-Martínez, Gema Moreno-Bueno

**Affiliations:** ^1^ Breast Cancer Department, MD Anderson Cancer Center, Madrid, Spain; ^2^ Centro de Investigación Biomédica en Red de Cáncer (CIBERONC), Madrid, Spain; ^3^ MD Anderson International Foundation, Madrid, Spain; ^4^ Pathology Department, Hospital Ramón y Cajal, Madrid, Spain; ^5^ Fundación Contigo Contra el Cáncer de la Mujer, Madrid, Spain; ^6^ Biochemistry Department, Universidad Autónoma de Madrid (UAM), Instituto de Investigaciones Biomédicas ‘Alberto Sols’ (CSIC-UAM), IdiPaz, Madrid, Spain

**Keywords:** leptin, leptin receptor, breast cancer, adaptive immunity, tumor-infiltrating lymphocytes (TILs)

## Abstract

Adipose tissue secretes various peptides, including leptin. This hormone acts through the leptin receptor (Ob-R), which is expressed ubiquitously on the surface of various cells, including breast cancer cells and immune cells. Increasing evidence points to an interaction between the tumor microenvironment, tumor cells, and the immune system. Leptin plays an important role in breast cancer tumorigenesis and may be implicated in activation of the immune system. While breast cancer cannot be considered an immunogenic cancer, the triple-negative subtype is an exception. Specific immune cells - tumor infiltrating lymphocytes - are involved in the immune response and act as predictive and prognostic factors in certain breast cancer subtypes. The aim of this article is to review the interaction between adipose tissue, through the expression of leptin and its receptor, and the adaptive immune system in breast cancer.

## Introduction

Overweight and obesity are considered to be pandemic in 2021. According to the World Health Organization, over 1.3 billion adults have a body mass index (BMI) ≥25 kg/m^2^; of these, 600 million are obese (1). These dramatic figures increased steadily throughout the world during the COVID lockdown (2, 3).

Overweight and obesity display a controversial relationship with breast cancer, which is highly dependent on menopausal status (4). For many years, little attention has been paid to the microenvironment of breast cancer, especially adipose tissue, which is considered an inert organ whose function is limited to storing fat. Adipose tissue produces a series of hormones, including adipokines. One of the most relevant adipokines is leptin, which is closely associated with breast cancer. Leptin not only controls energy balance, but is also associated with the neurological, hematopoietic, endocrine, reproductive, and immune systems (5). Together with cells from the innate and adaptive immune systems and cytokines (e.g., tumor necrosis factor alpha [TNFα] and interleukin [IL]-6), leptin can generate a pro-inflammatory stage in the tumor microenvironment.

Overall, breast cancer is considered a moderate immunogenic disease; however, the immune landscape of breast cancer is very heterogeneous and highly dependent on breast cancer subtype (6). Tumoral immune cell infiltration is predictive and prognostic in some breast cancer subtypes such as human epidermal growth factor receptor 2 (HER2)+ and triple negative. The importance of the characterization of the infiltrating immune cells (B-cell, T-cell, natural killer [NK] cell, etc.) is still being determined; however, breast tumors with higher tumor-infiltrating lymphocytes (TILs) are more responsive to treatments (e.g., immunotherapy, chemotherapy, radiation) and have better outcome than those with low TILs (7–9). However, the fact that breast cancer is not a very immunogenic tumor raises the question of what factors cause these TILs to be attracted around the tumor. In this sense, as explained above, the leptin axis could be one of the factors involved.

The present review analyzes the role of leptin with respect to the immune system in breast cancer, mainly its relation to the activation of TILs and puts forward a new hypothesis on the association between the two.

## Leptin and the Leptin Receptor (Ob-R) in Breast Cancer

Leptin is produced mainly by mature adipocytes. It is the protein resulting from the *ob* gene, which was identified in 1994. Mice with a mutation in the *ob* gene are obese, diabetic, and infertile because they do not produce leptin, which regulates food intake and body weight. Leptin is a non-glycosylated hormone containing 146 amino acids (10). It is characterized by a tertiary structure similar to that of members of the long-chain helical cytokine family (which includes IL-6, IL-11, IL-12, LIF, G-CSF, CNTF, and oncostatin M) (11).

Leptin acts mainly by maintaining energy homeostasis: in the obesity stage, high levels of circulating leptin generate negative feedback into a specific nucleus of the hypothalamus, the regulatory organ that increases sensitivity to the satiety signal. However, leptin is also characterized by systemic effects, including regulation of neuroendocrine, reproductive, hematopoietic, and immune functions (12–14). Serum leptin levels are directly related to adipose tissue mass and, hence, body mass index (BMI): obese people have higher leptin levels than lean people. Moreover, leptin levels are higher in women than in men, possibly because testosterone reduces leptin secretion, whereas estrogens increase its production (12, 15–17).

Leptin binds to the leptin receptor (Ob-R). Mice with a mutation in the *db* gene have a genetic deficiency in Ob-R resulting in the absence of expression of the long-form of Ob-R in all cell types (18–20). These mice are obese, diabetic, and infertile. Ob-R is a transmembrane receptor with a helical structure that is related to class I cytokine receptors (21). It is present in tissues in the pancreas, placenta, adrenal glands, stomach, hematopoietic cells, liver, heart, and lung, as well as in breast cells (22). Moreover, Ob-R is expressed ubiquitously on the surface of both peripheral and bone marrow–derived immune cells (23, 24). Ob-R has 6 isoforms resulting from alternative splicing of the gene: 4 have short cytoplasmic domains (Ob-Ra, Ob-Rc, Ob-Rd, and Ob-Rf), one is a long form (Ob-Rb), and the sixth is a soluble form, Ob-Re, whose main function is to control serum leptin levels. These 6 isoforms share an extracellular domain with common leptin-binding capacity, although their intracellular domains are different. The main effects of leptin on energy homeostasis and other metabolic functions are induced by the long isoform Ob-Rb (25).

In breast cancer, leptin expression is significantly correlated with that of Ob-R (13, 26). Of note, leptin and Ob-R are overexpressed in breast cancer epithelial cells compared with non-cancer mammary epithelial cells (13, 26). In fact, in approximately 92% of breast cancer cases, most of the carcinoma cells showed overexpression of leptin, as seen in the intensity of staining, which was as marked as for adipocytes. Ob-R was also expressed in 83% of the carcinoma cells.

The fact that Ob-R expression differs between healthy and cancerous tissue is of major importance since this difference could be used as a prognostic and/or predictive biomarker. Moreover, both factors (ligand and receptor) at the protein level might be prognostic factors in breast cancer (26). Recently, we showed Ob-R to be a predictive factor of pathological complete response in 100 early breast tumors managed with preoperative chemotherapy regardless of the molecular subtype (27). In our study, Ob-R was significantly expressed in HER2 and triple-negative breast cancer, younger patients, and patients with BMI ≥25 kg/m^2^ (27). As for expression of Ob-R in breast cancer subtypes, our results differ from those reported elsewhere (28–30), probably because of differences in the score and methodology used to evaluate Ob-R expression: one study used a score of 5% for positivity while in our study the score was 50% and, on the other hand, some studies analyzed Ob-R in cell lines while our study assessed breast tumors directly.

No data have been reported to date on the simultaneous expression of leptin and Ob-R in breast cancer or on the correlation with blood leptin levels. However, indirect data suggest a correlation between these 3 factors in breast cancer (31, 32).

**Figure 1:**
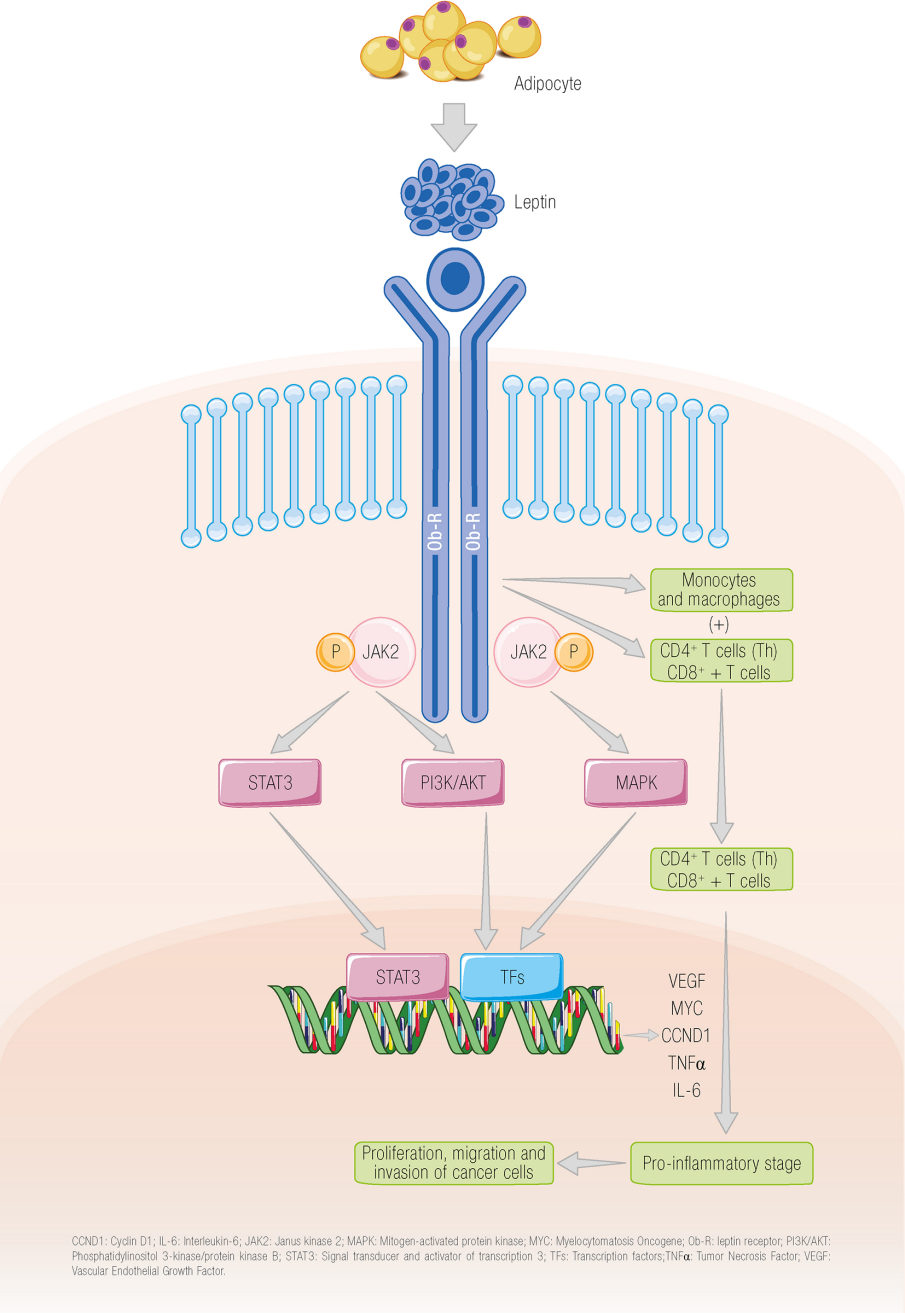
The binding of leptin to its receptor leads to the activation of different signaling pathways. Leptin binds to its receptor on the tumor cell, promoting the activation of different signaling pathways that, in turn, lead to the proliferation and invasion of tumor cells. The leptin receptor can also be located on different cells of the adaptive system leading to activation of these cells (34–37). CCND1, cyclin D1; IL-6, interleukin 6; JAK2; Janus kinase 2; MAPK, mitogen-activated protein kinase; MYC, myelocytomatosis oncogene; Ob-R, leptin receptor; PI3K/AKT, phosphatidylinositol 3 kinase/protein kinase B; STAT3, signal transducer and activator of transcription 3; TFs, transcription factors; TNFα, tumor necrosis factor alpha; VEGF, vascular endothelial growth factor. García-Estévez L ^©^.

Stimulation of the long receptor isoform by leptin leads to phosphorylation of Janus kinase 2 (JAK2), followed by phosphorylation of tyrosine residues 985 and 1138, which activate a series of pathways, namely, phosphatidylinositol 3–kinase–protein kinase B (PI3K/AKT), mitogen-activated protein kinase (MAPK), and signal transducer and activator of transcription 3 (STAT3) (33). Because of the activation of these signaling pathways, the leptin–Ob-R axis increases the proliferation, migration, and invasion of cancer cells (34–37) and to contribute to the release of vascular endothelial growth factor (38) (**Figure 1**).

Leptin appears to be a key driver in breast cancer tumorigenesis. The development and progression of a breast tumor is a long process of continuous interaction between the tumor cell and its surrounding microenvironment (39). The breast microenvironment is composed of mesenchymal cells (such as fibroblasts, adipocytes, blood cells, and leukocytes) and components of the extracellular matrix (including laminin, fibronectin, collagen, and proteoglycans). These elements play a role in mammary epithelial cell growth and differentiation. An increasing body of evidence shows that leptin enhances cell growth in both normal and malignant breast epithelial cells by activating various signaling pathways, such as those involving MAPK, JAK2–STAT3, and PI3K–AKT (22, 40). Furthermore, leptin has a growth-stimulating effect on both estrogen receptor–positive breast cancer (MCF7, T47D, MDAMB361) and estrogen receptor–negative breast cancer (MDAMB231, SKBR3) cell lines (41). Studies in animal models using mice with genetically obese leptin-deficient *Lep ^ob/ob^* or leptin receptor–deficient *Ob-R ^db/db^* did not detect mammary tumors in either case, indicating that an intact leptin axis is essential for these tumors to develop (42, 43).

## Immunological Landscape in Cancer and in Breast Cancer

The immune system can both inhibit and promote tumor expansion through a process known as “cancer immunoediting”, which comprises 3 phases, namely, tumor elimination, equilibrium (dormancy), and escape from immune surveillance (44, 45). To a large extent, the process depends on the plethora of immune cells presented at a specific point. In the elimination phase, transformed cells are identified and destroyed by infiltration of effector cells in the innate and adaptive immune system, as well as through production of tumor-inhibiting cytokines. The escape phase is sustained by chronic tumor-promoting inflammation, which primarily involves immunosuppressive cells and soluble factors (46). The escape phase is now considered a complementary hallmark of cancer (47).

In healthy tissue, effective immune surveillance is based on a series of cells that collaborate to maintain a healthy status with an anti-inflammatory effect. These cells are M2 macrophages, regulatory T cells (Treg), T helper 2 cells (Th2), eosinophils, and B cells. IL-4 and IL-10 are the main cytokines secreted from these cells (48). By contrast, a pro-inflammatory stage elicits the presence of multiple pro-inflammatory immune cell types, such as M1 macrophages, T helper 1 cells (Th1), CD8+ T cells, neutrophils, mast cells, and B cells (49). TNFα and interferon γ (IFNγ) secreted from these immune cells activate M1 macrophages, leading to further secretion of inflammatory cytokines, such as interleukin-1 beta (IL1β), TNFα, IL-6, and monocyte chemoattractant protein 1 (MCP1). In 1863, Virchow first postulated that cancer originates at sites of chronic inflammation (50). Chronic inflammation can result from infection, obesity, alcohol consumption or tobacco. In breast cancer, obesity is possibly one of the conditions that has been most often linked to the development of chronic inflammation and, therefore, to the development of breast cancer. Obesity causes adipocytes to become hypertrophic and die, and this is a key event in the change to an obese adipose tissue microenvironment which is pro-inflammatory. Adipocyte death triggers innate immune responses which shift the immune milieu towards a pro-inflammatory state which is associated with the infiltration of leukocytes, including macrophages, as well as mast cells and CD8-positive T lymphocyte (51). The rupture of the cell membrane of adipocytes also promotes the release of diverse cellular content (lipids and cytokines as well as fatty acids, ATP, reactive oxygen species, cholesterol and nucleic acids) into the microenvironment (52). Free fatty acids (FFAs) stimulate multiple inflammatory signaling pathways, and activation of transcription factor NF-kB, a key mediator in the immune response and adipose inflammation (53). These signals enhance the recruitment and accumulation of macrophages in the tumor microenvironment. Most of the macrophages in the obese adipose tissue microenvironment participate in the development of adipocyte hypertrophy by encircling the dying adipocyte forming crown-like structures (CLS), a hallmark of inflammation in adipose tissue (54). Macrophages are the main source of type 1 cytokines (e.g. TNF-α, IFN-γ, IL-1b and IL-6) as well as pro-inflammatory immune cells (granulocytes, group 1 innate lymphoid cells, B cells and CD8+ T cells), which act by preserving a state of chronic inflammation.

Breast cancer frequently arises as a result of genetic and epigenetic changes in genes that regulate the function of the mammary epithelial cells (55). Development of breast cancer can be prevented *via* induction of senescence or apoptosis of neoplastic cells by intrinsic tumor suppressive mechanisms (56). Similarly, the immune system is considered an extrinsic tumor suppressor capable of eliminating epithelial cells that have become breast cancer cells and limiting their growth when they have escaped intrinsic tumor-suppressive mechanisms (46, 57).

There are 2 types of immune system: the innate immune system and the adaptive immune system. These immune systems can be considered to be universal in all tumors, including breast cancer. The innate immune system is the first line of attack or, in other words, the recognition of tumor cells and their elimination. This innate system comprises myeloid-derived suppressor cells (MDSCs), mast cells, dendritic cells (DCs) and natural killer (NK) cells (58). MDSCs are a heterogeneous population comprising precursors of the myeloid-cell lineage. MDSCs may be broadly classified as monocytic and polymorphonuclear granulocytic subtypes (M-MDSCs and PMN-MDSCs, respectively) based on differences in the expression of their cell surface markers. Tumor-produced growth factors increase the generation of M-MDSCs and PMN-MDSCs, recruit these cells from bone marrow to solid tumors, and sustain their levels in blood. However, once in the tumor microenvironment, most M-MDSCs differentiate into immune-suppressive tumor-associated macrophages (59, 60). It has yet to be resolved whether PMN-MDSCs are a type of neutrophil or a distinct granulocyte population (58, 61). In the cancer microenvironment, tissue-resident macrophages and mast cells locally release soluble factors such as cytokines (IL-12 and IL-15 and type 1 interferon), bioactive mediators, chemokines and matrix-remodeling proteins that recruit additional leukocytes from the blood into damaged tissue (62–65). Recruited innate immune cells can directly act as the first line of attack *in situ*. At the same time, DCs transport foreign antigens (including tumor antigens) and migrate to lymphoid organs, where they present their antigens to adaptive immune cells. At this moment, cells, such as T lymphocytes or B lymphocytes, undergo clonal expansion in order to produce an `adaptive’ response targeted against the foreign agent (66, 67).

In breast cancer, the cellular network comprising the innate immune system plays a vital role in antitumor immunity through direct tumor killing as well as initiating, supporting and eliciting the adaptive immune response through secreted cytokines (68).

On the other hand, the adaptive immune system comprises 2 main mechanisms of action: humoral and cellular immunity. B cells are intimately involved in antibody-mediated humoral immune responses. By contrast, CD8+ CTLs (cytotoxic T lymphocytes) and NK cells are the primary effector immune cells that eliminate cancer cells. The intensity and composition of these elements differs across various types of cancer. For example, immune infiltrates are more extensive in melanoma, renal cell, lung, and colorectal cancer than other types of cancers, such as breast or prostate carcinoma (69).

CTLs can be induced to target specific antigens expressed on breast cancer cells (70–75). In The Cancer Genome Atlas, however, the correlation between increasing tumor mutational burden and expression of T-cell effector function varied substantially, and was found to be weakly positive in breast cancer (76). Studies in melanoma reveal few mutations to be immunogenic and, hence, capable of activating T-cell clones (51, 77). Nevertheless, the exact mechanisms by which these lymphocytes are mobilized around tumor cells remain unknown.

## The Role of Tumor-Infiltrating Lymphocytes (TILs) in Breast Cancer

Of all the cells that are part of the immune landscape of breast cancer, lymphocytes, or more specifically, tumor-infiltrating lymphocytes (TILs), are probably the most frequently studied and most relevant. TILs in breast cancer mainly comprise cytotoxic (CD8+) T cells, varying proportions of helper (CD4+) T cells and CD19+ B cells and, more rarely, NK cells (78, 79).

Stromal TILs, defined as the percentage of the stromal area infiltrated by TILs that are not in direct contact with carcinoma cells, are recommended for prognostic or predictive analyses (80). In fact, stromal TILs function as prognostic and predictive factors in breast cancer (81, 82). However, stromal TILs are more frequent in triple-negative breast cancer (TNBC) and HER2–positive breast cancers than in estrogen receptor (ER)–positive breast tumors (83, 84). The authors of a large, pooled analysis found that higher levels of stromal TILs predict pathological complete response (pCR) to neoadjuvant chemotherapy in all molecular subtypes (TNBC, HER2-positive and ER-positive/HER2-negative), although survival has been reported to be greater only in TNBC and HER2-positive breast cancer (84).

With respect to the role of stromal TILs as a predictive factor for pCR, the recent study by Floris et al. (85) provides very interesting insights into the possible interaction between adipose tissue and the immune system. In this retrospective study, the authors evaluated the role of BMI in modifying the effect of stromal TILs to predict pCR in TNBC patients treated with neoadjuvant chemotherapy and explored the prognostic value of stromal TILs according to BMI. A total of 445 TNBC patients were evaluated retrospectively. Regression analysis showed the interaction between stromal TILs and BMI to be statistically significant when both stromal TILs and BMI were considered as categorical variables. Furthermore, by considering stromal TILs and BMI as continuous variables, a statistically significant interaction was observed (interaction term p=0.04). However, when the authors analyzed the association between stromal TILs and pCR according to BMI, they found that among patients with a high frequency of stromal TIL tumors (≥30%), lean patients had a significantly higher pCR rate, with 38/52 (73.1%, 95% CI = 61.0–85.1) patients having a pCR compared with only 21/47 (44.7%, 95% CI = 30.5–58.9) in heavier patients. These data are very interesting in terms of the relationship between adipose tissue and immune response, primarily with stromal TILs resulting in reduced sensitivity to chemotherapy. The authors did not provide explanations for this, although one possibility might be that adipose tissue interacts with these stromal TILs to make them less effective against the tumor. This hypothesis is supported by Wang et al. (86), who found that T cells in the tumor microenvironment of diet-induced obese (DIO) mice demonstrated features of exhaustion. Specifically, flow cytometry showed that DIO mice were characterized by substantially higher counts of tumor-infiltrating CD8^+^ T cells (CD8^+^ TILs) expressing PD-1, T-cell immunoglobulin mucin-domain containing 3 (Tim3), and lymphocyte activating gene 3 (Lag3), and lower proliferation by Ki67 than control mice. Similarly, leptin levels in DIO mice were elevated, corresponding to increased values for of PD-1 on CD8^+^ T cells. The study reinforces the surprisingly positive association between obesity and the efficacy of cancer immunotherapy reported elsewhere (87); although the relationship between immune checkpoint inhibitors and T cells demonstrating features of exhaustion is not totally clear, this situation of exhausted lymphocytes could be reversed by checkpoint inhibitors that reactivate the immune system.

### The Connection Between Leptin and the Immune Adaptive System

Leptin is a critical regulator of the immune system and probably a key link between nutritional status and optimization of the immune response. There has long been evidence that leptin deficiency (*ob/ob*) and leptin-receptor deficiency (*db/db*) in both mice and humans result in immune defects (88) characterized by decreased total T-cell count, decreased CD4^+^ T-cell count, and a shift from a Th1 phenotype toward a Th2 phenotype (anti-inflammatory stage). This, in turn, generates protection against certain forms of autoimmunity and increased susceptibility to intracellular infections (89). Furthermore, leptin receptor–deficient (db/db) mice experience thymic atrophy, suggesting a relevant role for leptin in the regulation of immune cells.

One of the first *in vitro* studies to evaluate the role of leptin in activating T cells was that by Lord et al. (90), who demonstrated that leptin improves CD4^+^ T-cell responses mainly by binding to its receptor on T cells, rather than as the result of a direct effect on the stimulator cell. However, based on T-cell culture, the authors suggested that T cells first need to be stimulated by major histocompatibility complex (MHC) stimulator cells in order to respond to leptin effects. Furthermore, incubation of CD4^+^ T cells with leptin in the absence of allogeneic stimulator cells did not increase proliferation; consequently, cognate recognition by the T-cell antigen receptor (TCR) seemed to be necessary before leptin could exert its effect. *In vitro* studies from Martín-Romero et al. (91) confirmed this hypothesis: leptin alone cannot activate T lymphocytes cells despite overexpression of Ob-R in these cells (CD4^+^ and CD8^+^). According to Martín-Romero et al., T cells need to be co-stimulated by a non-specific stimulus such as lectin phytohemagglutinin (PHA) L in order to be activated by leptin. On the other hand, leptin induced the production of IL-2 by CD4^+^ T cells, thus modulating T-helper activation toward the Th1 phenotype (pro-inflammatory) through stimulation of INF-γ synthesis (90).

Although much remains to be discovered about the relationship between the leptin axis and the immune system, a recent study by Rivadeneira et al. (92) sheds some light on this issue. The authors used oncolytic *vaccinia* virus to promote T-cell tumor infiltration in melanoma models; however, the virus produces the recruitment of new but metabolically dysfunctional CD8^+^ T cells. The problem was overcome by adding leptin in the microenvironment, thus leading to potent T-cell activation. Also noteworthy in the study by Rivadeneira et al. is higher expression of Ob-R in activated and exhausted T cells with high PD-1 and Tim-3 expression. In this way, leptin induces metabolic reprogramming in T cells, and tumor-infiltrating T cells bear its receptor. Hence, leptin could prove useful in terms of therapy as a metabolic modulator of the immune response.

Leptin can affect the responsiveness and function of Treg cells, which play a key role in controlling peripheral immune tolerance and are abundant in adipose tissue (93). The fact that leptin negatively affects the proliferation of Treg cells could explain why Treg numbers are reduced and their function is impaired in obese patients (92). The above mentioned alterations could increase counts of adaptive immune cells, such as CD8^+^ T cells and CD4^+^ T helper type 1 cells, which produce proinflammatory cytokines (94). However, the association between leptin and Treg cells in breast carcinoma remains unexplored (**Table 1**).

**Table 1:** Potential effects of leptin in adaptive immune cells.

Cell Type	Ob-R Expression	Cytokine	Effect	Stage
CD4+	Yes	IL-2, IFN-γ	-Activation* -Proliferation -Survival -Promotion a bias toward T helper 1-cell	Pro-inflammatory
CD8+	Yes	IL-2, IFN-γ	-Activation* -Proliferation -Survival	Pro-inflammatory
Tregs	Yes	IL-2	-Inhibition of proliferation	Pro-inflammatory

*Previous lectin phytohemagglutinin (PHA) or concanavalin A stimulation is required (90) Tregs, T regulatory cells.

**Figure 2:**
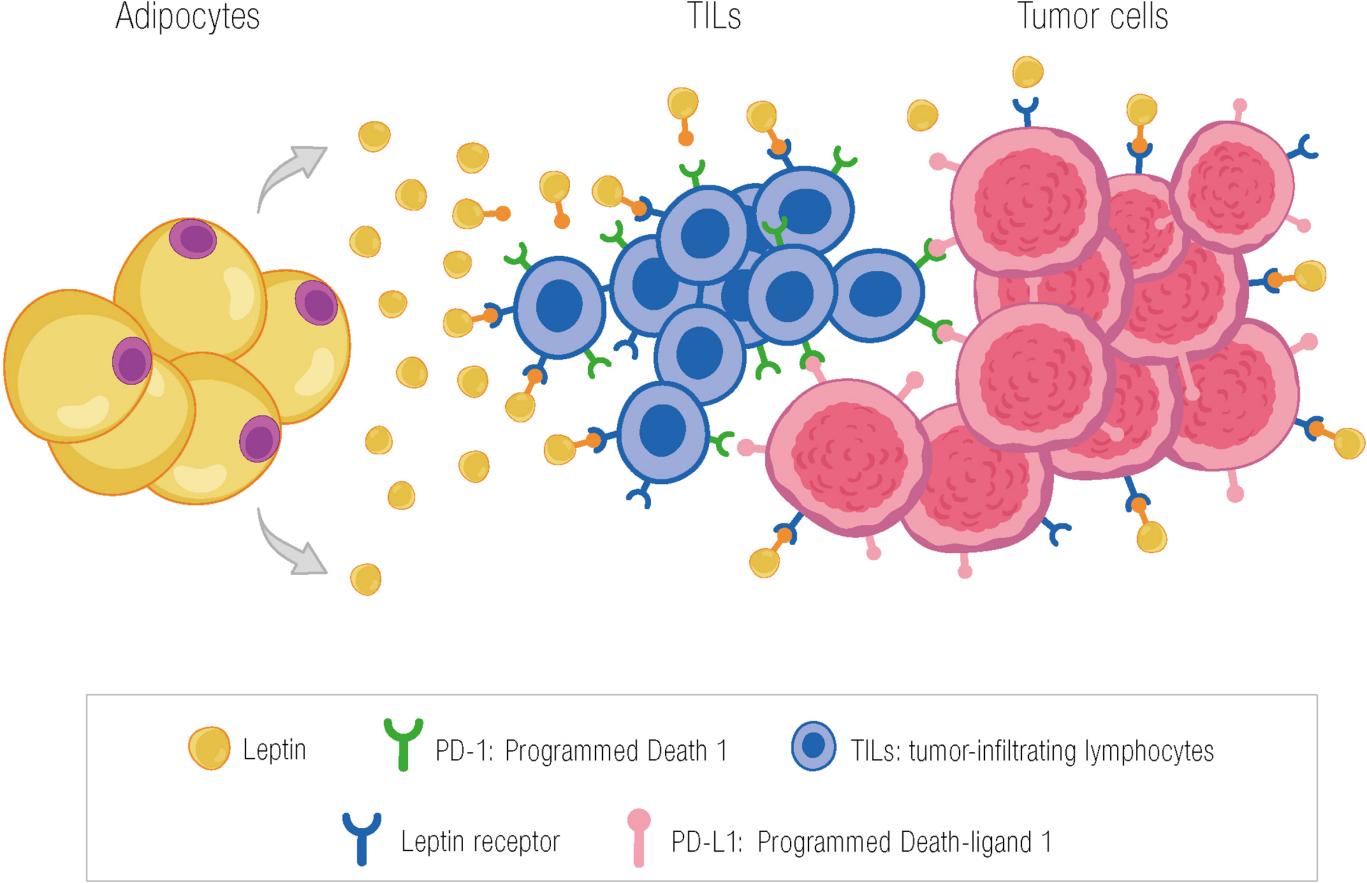
Potential effect of leptin in activating TILs in the tumor microenvironment through the leptin receptor. The presence of TILs in the tumor microenvironment may be due, among other factors, to the action of leptin through binding to its receptor on T cells. PD-1, programmed death 1; TILs, tumor-infiltrating lymphocytes; PD-L1, programmed death-ligand 1; García-Estévez L ^©^.

Most studies evaluating the role of leptin in the immune system are preclinical, with only limited clinical data available. Our group analyzed the association between Ob-R, TILs, and pCR in a group of 87 patients with breast cancer (luminal, triple-negative, and HER2 subtypes by immunohistochemistry) treated with neoadjuvant chemotherapy. We hypothesized that the leptin–Ob-R axis in breast cancer is involved in activating T cells. In fact, we found that Ob-R overexpression was generally associated with high TILs, although this finding was only significant in the HER2+ subtype (mean percentage of TILs in Ob-R–positive HER2+ tumors, 21.5% vs. 9% in Ob-R negative tumors; p=0.015). Furthermore, in patients with overexpressed Ob-R (>50% of positive cells with weak or strong staining), the mean percentage of TILs was significantly higher in tumors with pCR (responders) compared to tumors with residual disease (26.6% vs. 12.5%; p=0.005). Surprisingly, this difference was not observed in tumors with Ob-R that was not overexpressed, where the mean percentage of TILs did not differ between responders and non-responders (95). The next step would be to investigate the hypothesis that TIL populations might differ between tumors where Ob-R is overexpressed and those where it is not. It would also be interesting to find features of exhaustion that could be reversed by checkpoint inhibitors (**Figure 2**).

In our retrospective analysis, BMI was not correlated with TILs, in contrast with the study by Floris et al. (85). We observed a potential association between TILs and Ob-R in tumors with pCR. However, there are differences between the 2 studies; namely, the study by Floris et al. was conducted exclusively on TN tumors using BMI as an indicator of increased adipose tissue. In our study, the positive correlation between TILs and the leptin axis was found mainly in the HER2+ population and in tumors that achieve pCR. However, since our sample was small, our hypothesis must be confirmed with a larger sample. In any case, both studies highlight an interaction between adipocytes and T cells in breast cancer.

## Conclusions

Breast cancer is a complex disease in which both altered genes and the interaction between the tumor microenvironment and tumor cells are important events. The microenvironment comprises a plethora of cell types, including adipocytes. Long regarded as fat storage sites, adipocytes are now known to be very active and responsible for the release of a variety of adipokines. The high percentage of adipose tissue in mammary glands constitutes a microenvironment that enables the immune system to sustain immune responses (96, 97).

Expression of Ob-R by T and B cells is particularly interesting, since it points to the potential involvement of leptin in immune-cell activation and signal transduction. Analysis of this process could reveal new effects of leptin on as yet unexplored immune cell functions.

Preclinical and clinical breast cancer data already point to an interaction between adipose tissue (measured as BMI or *via* the leptin axis), the immune system, and the tumor. Given the presence of adipose tissue in the mammary gland, it is not unreasonable to hypothesize that there is an interaction between adipocytes and the substances they release and the immune response to the tumor. TILs are considered the most relevant part of the immune response currently evaluated in breast cancer; therefore, every attempt should be made to take advantage of their relationship with adipose tissue and, hence, leptin. Despite our lack of knowledge on the precise mechanisms of interaction involved in the disease, breast cancer remains a fascinating field of translational research.

## Author Contributions

LG-E and GM-B designed and wrote the manuscript. All authors provided editorial support and read the manuscript. All authors contributed to the article and approved the submitted version.

## Funding

The research carried out by our group (Annals of Oncology 2021) has been supported by a specific grant from the ACS Foundation. This study was partially supported by the Spanish Ministry of Economy and Innovation (PID2019-104644RB-I00 -GMB-), the Instituto de Salud Carlos III (CIBERONC, CB16/12/00295 –GMB-; partly supported by FEDER funds) and by the AECC Scientific Foundation (FC_AECC PROYE19036MOR -GMB-).

## Conflict of Interest

LG-E reports personal fees from Roche, Eisai, Daiichi Sankyo, and Palex.

The remaining authors declare that the research was conducted in the absence of any commercial or financial relationships that could be construed as a potential conflict of interest.

## Publisher’s Note

All claims expressed in this article are solely those of the authors and do not necessarily represent those of their affiliated organizations, or those of the publisher, the editors and the reviewers. Any product that may be evaluated in this article, or claim that may be made by its manufacturer, is not guaranteed or endorsed by the publisher.
